# Ten Cases of Excision of Joints

**Published:** 1873-03

**Authors:** Alfred T. Livingston

**Affiliations:** Buffalo, N. Y.


BUFFALO

### Medical and Surgical Journal.

VOL XII. MARCH, 1873. No. 8.

### Original Communications.

:o:

ART. I.—*Ten Cases of Excision of Joints; involving the Elbow, Wrist, Carpal, Phalangeal of Thumb, Hip, Knee and Tarsal Articulations.* By ALFRED T. LIVINGSTON, A. B.

A Thesis. Recommended for publication by the Faculty of the Buffalo Medical College.

The report of so many cases will necessarily occupy the full time proper to a paper of this kind, and I will therefore omit any general remarks upon the subject of of Excision of Joints, except such as may be suggested in connection with each case.

I am greatly indebted to Prof. Julius F. Miner for many interesting experiences in Surgery, and I deem it but just to make this acknowledgment here, since I have seen with him all the cases I am about to relate.

Beginning with the elbow joint, on which this operation is quite frequently, and of the larger joints, most successfully made, I will relate the history of

CASE I.

Daniel Shafer, aged 26, a German laborer from the oil regions of Pennsylvania, entered the Buffalo General Hospital November 26, 1871. Five weeks previously he had received a compound fracture of the right humerus near the elbow joint.

The arm was much swollen about the joint, anchylosis almost complete and suppuration was taking place through an opening just above the inner condyle of the humerus.

Thirteen years before, the patient had a simple fracture at nearly the same situation, and since then has had partial anchylosis of the elbow joint and a false joint at seat of fracture.

Examination by means of the probe discovered a mass of rough and softened bone.

Dr. Miner operated on this patient December 9, 1871, before the class, removing about six inches of the lower end of the humerus. The incision was made along the back of the arm and the bone carefully enucleated, separating it from its periosteum as thoroughly as possible.

The ulnar and radial surfaces of the joint, presenting no sign of disease, were not removed. The dressings were warm, either water alone or a weak solution of carbolic acid in that menstruum. The process of repair progressed rapidly and on February 7, 1872, the patient was discharged, the wound being entirely healed.

It is to be regretted that, since the latter date, the patient has been lost sight of, but before leaving the hospital he had so good use of his arm that he frequently carried a pail of water in the corresponding hand. The arm shortened between two and two and one-half inches, the remaining space, formerly occupied, by the bone, being filled with a cartilaginous substance. This, it was expected, would become ossified, but not knowing the whereabouts of the patient it cannot be determined.

The extent of the disease as seen after macerating the removed bone and the brief time that had elapsed since the injury, showed its progress to be very rapid and the operation most timely.

The second fracture was inter-condyloid, but how it was produced is not known.

Great care was taken, in peeling out the bone, not to injure any of the arteries that run along near its surface.

This is always to be observed and particularly in excisions of the humerus and the blade of the knife seldom used.

The periosteum should be separated from the bone, in all excisions, as completely as possible, and left intact, for on its presence depends in a great degree the reformation of osseous tissue. So much value, in fact, is put upon the preservation of the periosteum that M. Ollier claims to have demonstrated that an entire new bone may be reformed in all particulars like the original one, even to tubercles, tuberosities, etc , provided the periosteum be preserved entire.

CASE II.—INVOLVING ELBOW AND WRIST.

Miss Nellie B., of Utica, N. Y., aged 13, previously healthy, had a violent attack of inflammatory rheumatism (?) about the first of May last; its seat being the left elbow joint.

"The attack was unusually severe—the part swollen, very red and extremely tender and painful; pulse 130 to 140 per minute; temperature 105° Fahrenheit, with characteristic sweating," &c.

Dr. Daniel P. Bissell, to whom I am indebted for the history of the patient previous to and since her visit to Buffalo, treated her with cathartics, veratrum, colchicum, alkalies, &c., and the various symptoms subsided, leaving her quite comfortable.

A few days later the left wrist was similarly, but less severely, affected. This soon disappeared and her general health was quite good.

Early in June, the same arm became inflamed about two inches below the elbow joint; suppuration followed and tolerably healthy pus was discharged until the middle of July, when she came to Buffalo to be treated by Dr. Miner.

On the twenty-eighth of July, after probing the sinus through which the discharge took place and discovering carious bone, the Doctor made an incision along the inner side of the ulna beginning at the elbow joint and, extending it downwards as he discovered the further extension of the disease, ending it only when the wrist joint was reached. About half an inch of the lower extremity of the ulna was the only portion of the entire bone that did not present the appearance of disease; and this, upon subsequent maceration separated from the shaft showing it to be the epiphysis.

Dr. Miner removed the entire ulna, separating it from its periosteum, which was left almost complete. He also removed about an inch of the lower end of the humerus and about two inches of the upper end of the radius; these portions being also diseased.

All this was done without the ligation of any vessel.

The lips of this long incision were brought together by two or three stitches and a few adhesive straps lightly or rather loosely applied. Warm water dressings were used and arm laid upon a pillow. Two weeks from the day of the operation the patient visited Dr. Miner at his office, and a week or ten days later returned to her home.

A recent letter from Dr. Bissell informs me that there is still a small opening at the elbow from which a little pus is discharged, He "cannot find much, if any, ossific deposit in the bed of the ulna. She can flex and extend her fingers and partially rotate the hand. but does not flex the forearm without the aid of the other hand."

It will be observed that I have questioned the statement that the violent attack shortly preceding the occurrence of suppuration was inflammatory rheumatism, and I do so for this reason

Examination of the bone and portions of bones removed showed the existence of a central part, representing the original bone before disease, surrounded by a cylinder of bone of new growth varying from one-sixteenth to one-fourth of an inch in thickness. This central portion was necrosed; and the suppuration was the beginning of nature's effort to get rid of the dead and now foreign mass.

If the attack referred to be considered inflammatory rheumatism (the patient having previously been perfectly healthy and the only succeeding affection being the inflammation below the elbow, which was very quickly followed by suppuration) when shall we say this great change occurred which we have observed, viz., the death of a large amount of bone and the extensive deposit around it of new osseous material? It appears to me to be more in accordance with all the phenomena noticed to say that the attack was one of periostitis, or ostitis, or both, inducing the necrosis and deposit of new bone.

I have much faith in the final reproduction of a considerable portion of the ulna in this case, owing to the preservation of its periosteum.

This instance illustrates how rapid may be the progress of disease in bony structure, and how great an amount of such structure may be removed from a limb without destroying completely the usefulness of the limb.

CASE III.—INVOLVING THE ELBOW JOINT.

Daniel Kenan, aged six years, entered the Hospital of the Sisters of Charity January 24, 1872.

Some two years before he had been thrown from a sleigh, striking upon his left arm, bruising but not fracturing it.

Suppuration was occurring through a couple of sinuses above the elbow, and this had existed for a considerable period.

On the following day, after an examination with the probe which detected diseased bone, Dr. Miner removed, in the same manner as in Case I, the lower two-thirds of the humerus.

The articular surfaces of the ulna and radius were, here also, left in situ, no disease being apparent in them.

The little fellow progressed favorably, the incision and sinuses healed nicely and there was every indication of perfect success from the operation; but, before the poor child left the hospital he was attacked with the then prevailing epidemic, cerebro-spinal meningitis, and died from its effects.

The results, both in this and Case I, show that it is not requisite, as was supposed by the older surgeons, to remove all the surfaces of a joint when it is found necessary to remove a part of them.

The success attending each of these three cases of excision of the elbow joint gives additional proof to the already well-established value of the operation in cases of caries or necrosis involving this articulation.

### CASE IV.—INVOLVING THE WRIST JOINT.

Claus Von Schlausen, aged 13, had the metacarpal bones of the ring and middle fingers crushed with considerable laceration of the soft parts covering them.

Dr. Miner was called and removed the crushed bones together with the corresponding fingers, and also the *carpal bones* of the second row corresponding with the metacarpal bones removed. The sides of the hand were pressed together, so that there should not be a wide interval between the remaining fingers, and held by stitches and adhesive straps.

Union occurred and the boy has now a useful hand.

The removal of the carpal bones mentioned, which is rather a novel idea I think, was for the purpose of allowing the more complete approximation of the bases of the remaining metacarpal bones and of thus securing a more shapely and useful hand.

### CASE V.—INVOLVING THE PHALANGEAL ARTICULATION OF THE THUMB.

Michael O'Keif, aged 45, came to the hospital, December 13, 1872, with a crushed thumb.

It was very greatly swollen, the soft parts much contused and presenting a gangrenous appearance. In almost any mind except Dr. Miner's there would have been but one decision—amputation. But the Doctor, who is notably conservative in all cases, has a decided conservative *penchant* in dealing with injuries to the thumb. He therefore retained that organ in this instance; removing only the portions of bone that were crushed and that would surely have necrosed if allowed to remain. The distal extremity of the first, and the proximal extremity of the second, phalanx were removed.

Warm water dressings were employed, and, by this means, a better circulation was induced. The thumb was saved and, though a stiff one, is immensely better than no thumb at all.

In his lectures upon amputations and excisions, Dr. Miner dwells very emphatically upon the importance of preserving the thumb; it being supplementary or opposed, in its function, to the rest of the hand. In this teaching he appears to have anticipated other surgeons.

### CASE VI.—INVOLVING THE HIP JOINT.

Edward Bowles, aged 20, residing at Lancaster, N. Y., was afflicted with Morbus Coxarii on the right side.

On the invitation of Dr. Miner, I accompanied him, June 13, 1872, to the residence of the patient and assisted him in removing the head, neck and upper three inches of the shaft of the right femur.

An abundant and constant purulent discharge took place through a couple of sinuses which opened externally below Pouparts ligament.

The finger, passed through the larger of the two openings, discovered the head of the bone occupying the obturator foramen or pressing upon the surface its membrane. All the upper part of the femur was bared of periosteum and roughened by the disease.

The patient was in a very low condition—as pale, anæmic and emaciated as possible, pulse 120, and death seemed imminent, indeed, was daily expected by his friends.

The operation was made, not with any hope, on the part of the Doctor, of a cure, but, with the thought that the removal of the *cause* of so great a drain upon the system might give him relief and lengthen his life somewhat.

This operation more than sustained the prognosis as the patient survived it nearly three months. Under the circumstances it is of course no test of the value of the operation in this disease.

I ought perhaps say that this is Dr. Miner's first unsuccessful case of excision of the Hip Joint. A noticable point in this case was the slight shock the operation gave to the system, although so greatly weakened, the symptoms after, being little, if any, more marked than before, it occurred.

### CASE VII.—INVOLVING THE KNEE JOINT.

Thomas A. Cobb, aged 42, acting as special police in a saloon on Canal Street, while running after a drunken man who had committed some offense, was shot by the latter, the pistol ball entering just below the left patella and, passing into the joint, cutting a groove along the articular surface of the tibia from before backward and lodging near its posterior edge. The exact position of the ball could not be determined before the operation as it could not be felt in the popliteal space nor by the probe, passed through the opening made by the ball; but as it was clearly shown from its course that it had passed into the Joint, the question of amputation or excision was discussed and excision decided upon.

Dr. Miner performed the operation, before the class, in the Hospital of the Sisters of Charity. He made a U flap extending from the condyle of the femur on either side as far down as the attachment of the quadriceps extensor tendon to the tibia. On raising the flap the Joint was exposed and the articulating surfaces of both tibia and femur were removed. The patella was also dissected out of the flap.

The wound was left open to give free exit to pus.

There was no tendency whatever to heal exhibited by the wound and the patient died two weeks after the operation with every indication of pyæmia.

The propriety of excision in gunshot injuries involving the knee is concurred in by Prof. Moore, in his Lectures.

During the recent civil war in this county eighteen cases of excision of this Joint occurred. Seventeen of these patients died. Previous to the war seven cases were reported, of which five died.

Prof. Hamilton thinks the average mortality following this operation in all cases to be at least 50 per cent. Other observers make it much less, as 25 to 30 per cent.

In gunshot injuries alone however the mortality is very great.

CASE VIII.—INVOLVING THE TARSAL ARTICULATIONS.

Dennis Gleason, aged 23, a native of Ireland, entered the Hospital of the Sisters of Charity on the 6th of June 1871.

About one year and a half previous to this he began having what he supposed to be rheumatism, in his right foot.

This continued until March, 1871, when inflammation occurred in the foot which was followed by suppuration.

I first saw him at one of the Surgical clinics of October, 1871. He was then extremely pale and anæmic and hardly appeared able to endure the operation proposed. There were openings on each side of the foot through which the discharge took place and, on passing the probe through these, much diseased bone was discovered.

Prof. Miner removed a considerable portion of the tarsal bones, including the scaphoid, cuboid and cuneiform bones, through openings made at either side of the foot by laying back triangular flaps. The space left by the removal of the bones has filled with a solid material, the external wounds have healed, the patient is looking and feeling quite well and but for one circumstance the result would be most satisfactory. During the process of repair, which occupied a long time, on account of low vitality of the system, the foot remained constantly in one position, viz., flexed at a little more than a right angle with the line of the leg and passive motion not having been used the ankle joint anchylosed, so that although the patient now walks about the Hospital every day, without a cane and without pain, he walks very awkwardly being obliged all the while to keep the right foot in advance of the other.

In all injuries involving or in the neighborhood of Joints, *passive motion* should be employed as early as the third week. If this is not practicable or if there is a probability that the Joint will anchylose, the limb should be given that form which, with the Joint stiff, will permit the greatest facility in locomotion.

This rule, of course, applies as well to operations as to accidental injuries.

### CASE IX.—INVOLVING THE TARSAL ARTICULATIONS.

Miss Mary M——, of Suspension Bridge, New York, aged 16, received a fall in February, 1872, which produced a considerable injury to her right foot. She was carried home and since the accident has been unable to walk without the aid of crutches. The foot became much swollen and suppuration followed.

In July last she came to the Hospital of the Sisters of Charity in this city and was examined by the surgeon then in charge, who discovered necrosed bones, but advised a delay of the operation until cold weather. She returned to the hospital the last of October and on November 6th, 1872, Dr. Miner removed the bones and portions of bones which he found diseased. The operation was entirely similar to the one last described, the bones removed being the same with the addition of the proximal extremities of most of the metatarsal bones. The foot shortened about an inch and the remainder of the space, left by the removal of the bones, filled with a mass of cartilaginous consistence. The patient returned to her home during the holidays with the foot entirely healed.

### CASE X.—INVOLVING THE TARSAL ARTICULATIONS.

Mrs. M——, of Sheffield, Pennsylvania, in June, 1872, by stepping upon a nail, drove it into the sole of her foot toward the outside. The result of this was caries of some of the bones of the foot.

The patient came to Buffalo to be treated by Prof. Miner, who operated upon her December 6, 1872, removing the cuboid, external cuneiform, the whole of the fifth metatarsal bone and such portions of the middle cuneiform and fourth metatarsal bones as were diseased. The wound healed nicely from the bottom and the patient was allowed to return to her home on the ninth day of January, 1873, being able to stand upon the affected side.

The last three cases described are interesting because they are operations not generally favored by surgeons, and in their design are highly conservative. For example Prof. Hamilton of New York, in his recent work on Surgery, speaks very discouragingly of excisions of the smaller tarsal bones for caries or necrosis. Of the scaphoid, he says "it is seldom the malady is presented in a sufficiently stationary form to warrant exsection;" of the cuneiform bones, he has "seen no examples of caries of these bones which would justify excision." His theory is that the disease extends by continuity along the synovial sacs which separate the bones, and so many of these smaller bones being covered more or less by the same synovial sac, disease in one of the bones will almost necessarily involve all.

The result in the cases I have related certainly does not appear to me to contra-indicate the practice of excisions in such conditions. I regret that the interval since the operations upon the last two patients is not greater, that we might, with still more complete assurance, decide upon their value. The present appearances are that they are in every respect successful; and of the value of the operation in Case VIII., there is not the least doubt.

BUFFALO, N. Y., January 15, 1873.

